# Physical and brain frailty in ischaemic stroke or TIA: Shared occurrence and outcomes. A cohort study

**DOI:** 10.1177/23969873231186480

**Published:** 2023-07-07

**Authors:** Martin Taylor-Rowan, Melanie Hafdi, Bogna Drozdowska, Emma Elliott, Joanna Wardlaw, Terence J Quinn

**Affiliations:** 1 Institute of Health and Wellbeing, University of Glasgow, Glasgow, UK; 2 Department of Neurology, Amsterdam UMC, University of Amsterdam, Amsterdam, Netherlands; 3 Institute of Cardiovascular and Metabolic Sciences, University of Glasgow, Glasgow, UK; 4 Centre for Clinical Brain Sciences, Edinburgh Center in the UK Dementia Research Institute, University of Edinburgh, Edinburgh, UK

**Keywords:** Frailty, cognition, dementia, stroke

## Abstract

**Background:**

There is increasing interest in the concept of frailty in stroke, including both physical frailty and imaging-evidence of brain frailty. We aimed to establish the prevalence of brain frailty in stroke survivors as well as the concurrent and predictive validity of various frailty measures against long-term cognitive outcomes.

**Methods:**

We included consecutively admitted stroke or transient ischaemic attack (TIA) survivors from participating stroke centres. Baseline CT scans were used to generate an overall brain frailty score for each participant. We measured frailty via the Rockwood frailty index, and a Fried frailty screening tool. Presence of major or minor neurocognitive disorder at 18-months following stroke or TIA was established via a multicomponent assessment. Prevalence of brain frailty was established based upon observed percentages within groups defined by frailty status (robust, pre-frail, frail). We assessed the concurrent validity of brain frailty and frailty scales via Spearman’s rank correlation. We conducted multivariable logistic regression analyses, controlling for age, sex, baseline education and stroke severity, to evaluate association between each frailty measure and 18-month cognitive impairment.

**Results:**

Three-hundred-forty-one stroke survivors participated. Three-quarters of people who were frail had moderate-severe brain frailty and prevalence increased according to frailty status. Brain frailty was weakly correlated with Rockwood frailty (Rho: 0.336; *p* < 0.001) and with Fried frailty (Rho: 0.230; *p* < 0.001). Brain frailty (OR: 1.64, 95% CI = 1.17–2.32), Rockwood frailty (OR: 1.05, 95% CI = 1.02–1.08) and Fried frailty (OR: 1.93, 95% CI = 1.39–2.67) were each independently associated with cognitive impairment at 18 months following stroke.

**Conclusions:**

There appears to be value in the assessment of both physical and brain frailty in patients with ischaemic stroke and TIA. Both are associated with adverse cognitive outcomes and physical frailty remains important when assessing cognitive outcomes.

## Introduction

Frailty is a highly prevalent condition amongst stroke and transient ischaemic attack (TIA) survivors, existing in as many as 28% of patients upon admission to the acute stroke unit.^1,2^ Frailty assessment is increasingly being incorporated into acute care pathways but has yet to be routinely adopted in stroke.^2^ Evidence suggests there is prognostic value in assessing for frailty in stroke: delirium, mortality and post-stroke cognitive outcomes are all associated with frailty status.^3–5^

Frailty can be characterised as a state of multisystem physiological vulnerability with diminished capacity to manage external stressors.^6^ Operationalisation of frailty can be highly variable. Numerous frailty measurement methods exist, reflecting the uncertainty regarding the best way to assess this syndrome.^7^ However, although precise measurement can vary, two of the most commonly measured constructs include the frailty phenotype, as developed by Fried et al.,^8^ and accumulated deficits frailty, as developed by Rockwood et al.^9^ Fried frailty is typically assessed by measuring symptoms of physical frailty such as unexplained weight loss, muscle weakness, slow mobility, exhaustion, and low physical activity. Whereas Rockwood frailty is assessed via a more global measure of frailty that encapsulates a combination of physical, social and neuropsychological deficits that accumulate over the life-course.

Evidence of pre-existing damage visible on a brain scan at the time of the stroke, such as atrophy, leukoaraiosis, or old infarcts, can provide valuable prognostic information following stroke.^10^ However, generating a composite of this damage via a ‘brain frailty’ scale appears to provide stronger prognostic information than measuring each type of damage in isolation.^11^ In general, brain frailty is a state of reduced neurophysiological reserve and may predispose people to poor cognitive outcomes.^12^ Recently, Appleton et al.^11^ tested a scale for measuring brain imaging-based brain frailty^13^ that involves routinely observable neuroimaging markers (leukoaraiosis, cerebral atrophy and old infarcts). However, the scale has not yet been validated directly against measures of Fried or Rockwood frailty and we do not know the extent to which imaging-derived brain frailty co-occurs with Fried or Rockwood frailty.

Traditional measures of frailty, such as Fried or Rockwood, tend to be considered in isolation from directly observed imaging-derived brain frailty despite increasing evidence indicating they may be associated.^14,15^ If traditional measures of frailty are synonymous with brain frailty, then measuring each construct separately is inefficient. On the other hand, if they are distinct, establishing their independent associations with long-term cognitive outcomes could help improve clinical decision making.

## Aims

Our study had three main aims:

1) to validate a measure of imaging-derived brain frailty against traditional (Fried and Rockwood) measures of frailty.
2) to establish the prevalence of brain frailty in those who are/are not frail (as defined according to each traditional measure).
3) to examine the association between pre-stroke frailty and pre-stroke brain frailty with long-term cognitive impairment and dementia following stroke or TIA.

## Methods

We followed STROBE (Strengthening the Reporting of Observational Studies in Epidemiology) guidelines^16^ for reporting. This is a sub study of the APPLE (Assessing Psychological Problems in stroke: A longitudinal Evaluation; research registry ID: 1018) project—a multicentre, prospective longitudinal cohort study embedded within the UK National Health Service. A summary of our methodology is provided below. Comprehensive details are available in the study protocol.^17^ Ethical approval was approved by the Scotland A Research Ethics committee and obtained for all participating sites (REC number:Sixteen/SS/0105).

### Setting

APPLE recruited consecutively admitted stroke and transient ischaemic attack (TIA) survivors admitted to participating acute stroke centres (November 2016 to February 2019) with no exclusions based on age, stroke-type, stroke-severity, or comorbidity. Proxies were used to provide consent wherever participants were unable to consent themselves. This sub-study was restricted to sites with CT brain raw images available for analysis.

### Brain frailty assessment

Baseline CT brain scans were performed for all participants as part of routine acute care. We adopted an approach developed in IST-3^13^ and further refined for application,^11^ to generate a brain frailty score for each participant using baseline CT scan images. A detailed description of this approach can be seen in the protocol.^18^ In brief, two trained assessors (MH; MT), who were blinded to clinical details, rated baseline CT scans using a set proforma^18^ and scored presence of leukoaraiosis, cerebral atrophy, and old infarcts. Scans were rated against a standard template (Supplemental Materials Figure S1). ‘Modest’ leukoaraiosis, ‘modest’ atrophy (centrally and/or cortically) and presence of old focal cortical or subcortical vascular lesions were combined to generate a brain frailty score ranging from 0 (no brain frailty) to 3 (severe brain frailty).

### Frailty assessment

We measured frailty according to two of the most commonly employed frailty concepts: Rockwood’s ‘accumulated deficits’ global measure of frailty, and Fried’s ‘frailty phentoype’, which focuses on physical frailty.

Rockwood et al.^7^ frailty was measured via a 32-item frailty index (Supplemental Materials Figure S2), constructed based on previously validated frailty indexes and/or recommended guidelines.^19^ A combination of participant medical records and self/informant reported functional measures^20^ and Independent Activities of Daily Living^21^ were used to identify physical, functional, cognitive or psychological issues present before the stroke (informant reports were only used in cases where the participant was not able to complete a self-reported measure). Possible scores ranged from 0 to 100, with scores closer to 100 suggesting greater frailty. Participants were categorised as ‘robust’, ‘pre-frail’ and ‘frail’ using recommended cut-points of <8; 8–24 and >24, respectively.

Fried frailty was established via a self-report frailty screening tool.^22^ Where possible, informant reports were used when self-reported information was unavailable. The measure involves self-reported symptoms of physical frailty including exhaustion, unexplained weight loss, and low physical activity. Cut-points recommended by the scale developers were used to identify ‘frail’ and ‘non-frail’ participants.

### Outcome assessment

We employed a centrally adjudicated approach to establish presence of major and minor cognitive impairment at 18 months following stroke. Participants received a multi-domain cognitive assessment battery (details are proved in the APPLE protocol^17^) at baseline, 1 month, 6, 12 and 18 months. Performance on cognitive assessment was supplemented by evaluation of clinical case notes and other medical records up to an 18-month follow-up timeframe. Two assessors (medical and psychology backgrounds) independently evaluated the available data for each participant, and a consensus diagnosis, based on Diagnostic and Statistical Manual (DSM5) criteria,^23^ was established. Cognitive impairment diagnoses were applied conservatively; only formal diagnoses of major or minor neurocognitive impairment established via a medical professional, or consistent performance on cognitive testing that fell below standardised thresholds (e.g. performance below standardised cut points for impairment on the abbreviated mental test, and/or performance at 1.5 SD below age-matched norms on respective neuropsychological tests) for impairment were adjudicated as impaired at 18-months. All adjudications were made without knowledge of participants’ frailty or brain frailty status.

### Statistical analyses

Cohen’s weighted Kappa was used to establish inter-observer reliability of brain frailty ratings.

We conducted Spearman’s rank correlation to evaluate the concurrent validity of brain frailty relative to traditional frailty measures. The correlation coefficients were interpreted using conventional cut-points.^24^

Prevalence of brain frailty was established based upon observed percentages within the study population. Pre-specified cut-offs were applied to the Rockwood frailty index to differentiate ‘frail’, pre-frail and ‘non-frail’ participants. Fried frailty was established via dichotomisation at the recommended cut point.^22^ Confidence intervals were generated for each prevalence estimate.^25^

Multivariable logistic regression analysis was preformed to investigate the association between brain, physical and global measures of frailty with long-term cognitive impairment 18-months following stroke. Primary analysis (Model 1) controlled for age, sex, stroke severity and years in education. Brain frailty and Fried frailty were analysed on an ordinal scale, while Rockwood frailty was analysed on a linear scale.

We performed a sensitivity analysis restricted to participants with 18-month cognitive data only to ensure that our analysis was not overly influenced by adjudicated outcomes that relied more heavily on clinical case notes for 18-month diagnoses.

We conducted a series of subgroup analyses. In subgroup analysis 1, we excluded participants who had a known diagnosis of dementia at baseline in order to improve the clinical applicability of our analyses. In subgroup analysis 2, we additionally excluded people who had a TIA only.

We also conducted post hoc analyses to (1) explore if brain frailty was associated with long-term cognitive impairment independent of Fried or Rockwood frailty (Model 2), and vice-versa; (2) explore if brain, Fried or Rockwood frailty was associated with long-term cognitive impairment independent of baseline cognitive test scores – measured via the abbreviated mental test-plus (AMT-plus – an amalgamation of brief cognitive screening tests with overlapping assessments questions. Details of specific test components can be seen in study protocol^17^) (Model 3); (3) explore if brain frailty was associated with long-term cognitive impairment over both baseline cognitive scores and frailty scores, or if Fried or Rockwood measures of frailty were associated with long-term cognitive impairment over both baseline cognitive scores and brain frailty scores (Model 4).

A univariate cox regression survival analysis was performed to investigate if there was a difference between brain, Fried or Rockwood frailty status and likelihood of death during follow-up.

Regression analyses used a complete case approach. We conducted univariate logistic regression analyses to investigate differences between brain, Fried or Rockwood frailty status and missing data. Statistical assumptions were checked for each analysis. SPSS version 27 (IBM) was used for all analyses.

## Results

**Figure 1.**
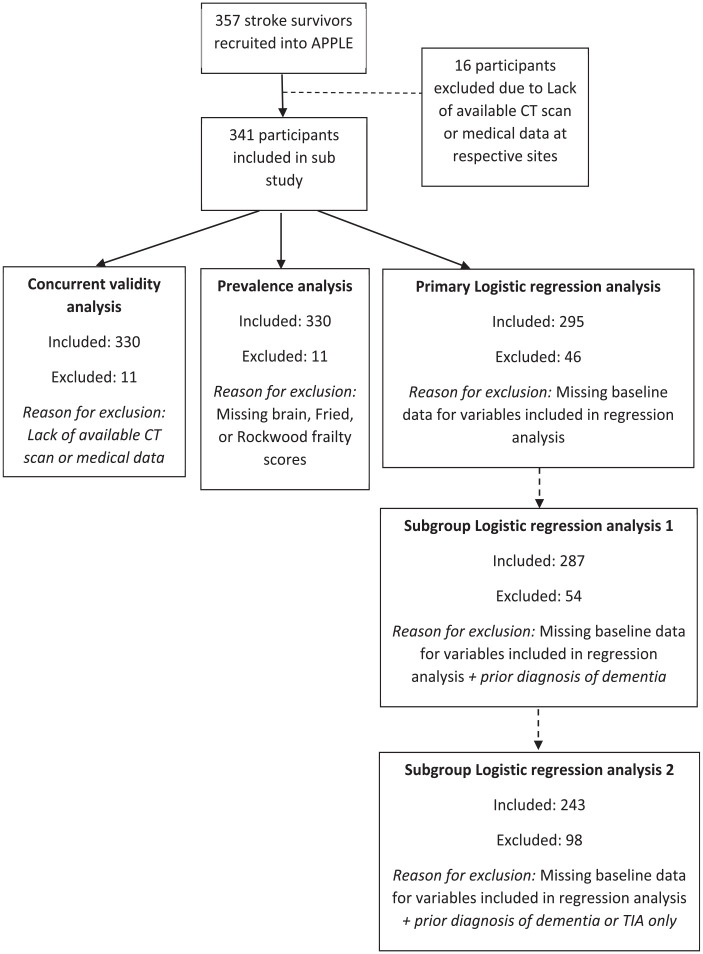
Flow chart of study attrition.

Of 357 participants in APPLE, 341 were recruited from sites with CT brain data available (Figure 1 and Table 1).

**Table 1.** Baseline study population characteristics.

Variable	Overall (*N* = 341)*
Age (Median; 25th–75th percentile)	71 (60–80)
Sex Male (%)	188/341 (55.1)
Stroke-type (%)	
Total anterior circulation stroke	27/336 (8.0)
Partial anterior circulation stroke	115/336 (34.2)
Lacunar stroke	82/336 (24.4)
Posterior circulation stroke	66/336 (19.6)
Transient ischaemic attack	46/336 (13.7)
NIHSS (Median; 25th–75th percentile)	2 (1–4)
Pre-stroke modified Rankin Scale (nn; %)	
0–2	283/339 (83.4)
3–5	56/339 (16.5)
Vascular disease (nn; %)	102/341 (29.9)
Heart failure (nn; %)	26/341 (7.6)
Post-stroke delirium (nn; %)	3/339 (0.9)
Post-stroke aphasia (nn; %)	46/339 (13.6)
Previous stroke/TIA (nn; %)	86/341 (25.2)
Diabetes mellitus (nn; %)	82/341 (24.0)
Atrial fibrillation (nn; %)	56/341 (16.4)
Alcohol dependency (nn; %)	36/341 (10.6)
Education years (Mean; SD)	11.9 (3.4)

* Some denominators ≠ 341 due to missing data.

Three-hundred-thirty-two participants had baseline CT scan data available, 329 had Fried phenotype frailty scores available, and 339 had a Rockwood frailty index score. Thirty-three participants had missing clinical or demographic baseline data and 22 participants had missing baseline cognitive data. Follow-up cognitive test data was available for 266 (78%) participants at at least one time-point after baseline; 160 out of 308 (52%) surviving participants had 18-month cognitive test data available; secondary and primary care follow-up data were available for all participants (Figure 1).

Univariate regression analysis showed that brain frailty (OR: 1.38, 95% CI = 0.95–2.02) and Rockwood frailty scores (OR: 1.02, 95% CI = 0.98–1.06) were not associated with missing baseline data; but higher Fried frailty scores were associated with missing baseline data (OR: 1.64, 95% CI = 1.12–2.41). Both brain frailty (OR: 1.31, 95% CI = 1.05–1.64), and Rockwood frailty (OR: 1.04, 95% CI = 1.02–1.07) were associated with missing 18-month cognitive data but not Fried frailty scores (OR: 1.15, 95% CI = 0.90–1.46).

Thirty-three (10%) participants died before end of study (18-months post-baseline); survival analysis indicated each point increase of brain frailty (HR:1.84, 95% CI = 1.29–2.62), Rockwood frailty (HR: 1.06, 95% CI = 1.04–1.09), and Fried frailty (HR: 1.59, 95% CI = 1.14–2.22) significantly increased the risk of death before end of study.

Inter-observer reliability of brain frailty ratings was acceptable (weighted kappa = 0.76).

### Concurrent validity of brain frailty scale

**Figure 2.**
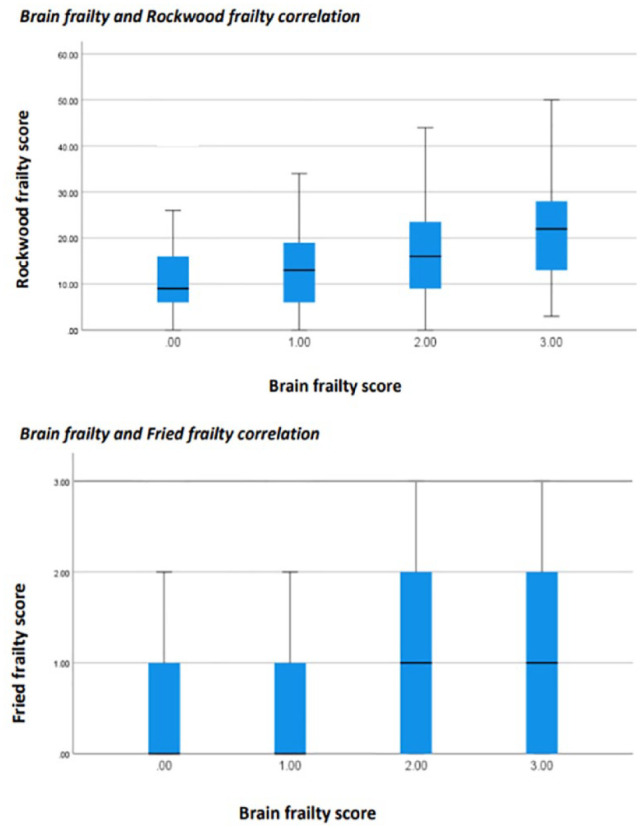
Correlation between brain frailty and traditional frailty measures.

Spearman’s rank suggested a significant, weak correlation between brain frailty and Rockwood frailty (rho: 0.336; *p* < 0.001). There was also a weak correlation between brain frailty and physical frailty (rho: 0.230; *p* < 0.001)(Figure 2).

### Prevalence of brain frailty in those who are physically frail

**Figure 3.**
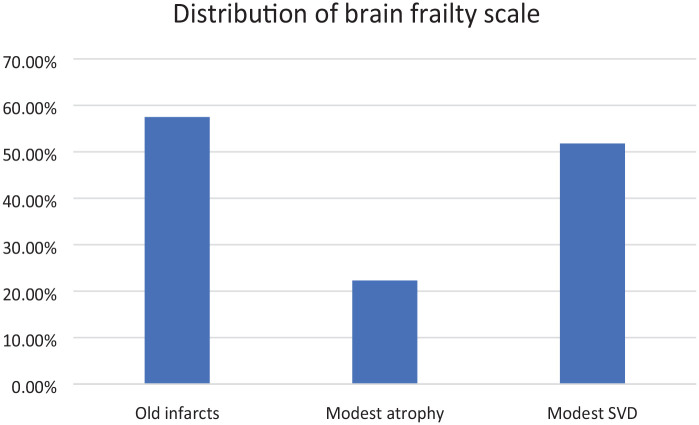
Distribution of brain frailty scale.

A total of 106/332 (32%, 95% CI = 27−37) participants had a brain frailty score of 1; 96/332 (29%, 95% CI = 24−34) had a score of 2; and 46/332 (14%, 95% CI = 10−18) had a score of 3. Distribution of each component of the brain frailty scale can be seen in Figure 3. Fifty-nine out of 330 (18%; 95% CI = 14−22) participants were categorised as frail (cut point > 0.24), 172/330 (52%, 95% CI = 47−58) were pre-frail (cut point 8–24) and 98/330 (30%, 95% CI = 25−35) were ‘robust’, according to the Rockwood frailty index (score < 8). One hundred seventy out of 320 (53%, 95% CI = 48−58) participants were classified as frail on the Fried frailty measure. Prevalence of brain frailty according to frailty status can be seen in Table 2.

**Table 2.** Prevalence of brain frailty according to frailty status.

	Rockwood frail	Rockwood Pre-frail	Rockwood robust	Fried frail	Fried robust
Brain frailty score 1 (nn; %)	12/59 (20%; 95% CI = 11−33)	54/172 (31%, 95% CI = 25−39)	40/98 (41%, 95% CI = 31−51)	47/170 (28%, 95% CI = 21−35)	57/150 (38%, 95% CI = 30−46)
Brain frailty score 2 (nn; %)	24/59 (41%; 95% CI = 28−54)	52/172 (30%, 95% CI = 23−38)	19/98 (19%, 95% CI = 12−29)	60/170 (35%, 95% CI = 28−43)	30/150 (17%, 95% CI = 14−27)
Brain frailty score 3 (nn; %)	19/59 (32%, 95% CI = 21−46)	19/172 (11%, 95% CI = 7−17)	7/98 (7%, 95% CI = 3−14)	30/170 (18%, 95% CI = 12−24)	14/150 (9%, 95% CI = 5−15)

**Figure 4.**
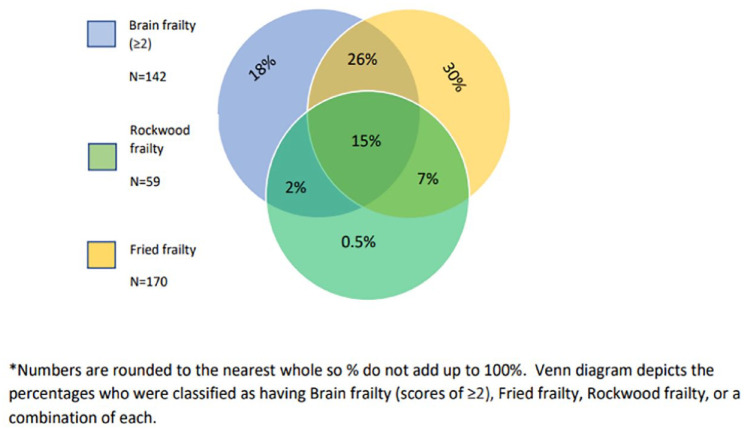
Overlap between participants categorised as frail according to each frailty measure.

Overlap between measures of frailty were limited: only 22% of participants were classified as frail on both the Rockwood and Fried measures. By comparison, 41% of participants were categorised as frail on both brain frailty and Fried scale, while 17% were classified as frail on both brain frailty and Rockwood scale. Degree of overlap for all three frailty measures is illustrated (Figure 4).

### Brain/physical frailty association with long-term cognitive impairment

Two hundred ninety-five participants were included in primary analysis; 69/295 (23.4%) had adjudicated cognitive impairment at 18 months. In Model 1, each point increase on the brain frailty scale (OR: 1.64, 95% CI = 1.17–2.32), Rockwood frailty index (OR: 1.05, 95% CI = 1.02–1.08), and Fried frailty scale (OR: 1.93, 95% CI = 1.39–2.67) independently increased odds of cognitive impairment 18 months after stroke. Model 2 suggested brain frailty was associated with long-term cognitive impairment independent of both Rockwood frailty (brain frailty OR: 1.47, 95% CI = 1.03–2.09) and Fried frailty (brain frailty OR: 1.48, 95% CI = 1.04–2.12). Rockwood frailty was similarly associated with long-term cognitive impairment independent of brain frailty (Rockwood OR: 1.04, 95% CI = 1.01–1.07), as was Fried frailty (Fried OR: 1.78, 95% CI = 1.27–2.49). In Model 3, which included adjustment for baseline cognitive status, brain frailty was no longer significantly associated with cognitive impairment (OR: 1.40, 95% CI = 0.93–2.10), while Rockwood frailty (OR: 1.04, 95% CI = 1.01–1.08) and Fried frailty (OR: 1.57, 95% CI = 1.08–2.26) remained significantly associated. Model 4, which included brain and Fried or Rockwood frailty controlled for baseline cognitive score, suggested Fried frailty remained significantly associated with long-term cognitive impairment independent of both brain frailty and baseline AMT-plus scores (Fried frailty OR: 1.49 95% CI = 1.03–2.18); however, Rockwood frailty (OR: 1.04, 95% CI = 0.99–1.076) and brain frailty (OR: 1.29, 95% CI = 0.85–1.95) were not (All models Table 3).

**Table 3.** Logistic regression output for models 1–4. Brain frailty regression output for association with 18-month cognition. Rockwood frailty regression output for association with 18-month cognition. Fried frailty regression output for association with 18-month cognition.

Characteristic	Multivariable model 1	Multivariable model 2	Multivariable model 3	Multivariable model 4
Age	OR: 1.05; 95% CI = 1.01–1.08	OR: 1.05; 95% CI = 1.01–1.08	OR: 1.05; 95% CI = 1.01–1.09	OR: 1.04; 95% CI = 1.01–1.08
Sex	OR: 0.75; 95% CI = 0.41–1.37	OR: 0.79; 95% CI = 0.43–1.46	OR: 0.79; 95% CI = 0.39–1.60	OR: 0.83; 95% CI = 0.41–1.69
Stroke severity	OR: 1.07; 95% CI = 1.00–1.15	OR: 1.05; 95% CI = 0.98–1.13	OR: 1.01; 95% CI = 0.91–1.14	OR: 0.99; 95% CI = 0.88–1.12
Education (years)	OR: 0.92; 95% CI = 0.82–1.03	OR: 0.93; 95% CI = 0.83–1.04	OR: 0.96; 95% CI = 0.86–1.08	OR: 0.97; 95% CI = 0.87–1.09
Brain Frailty	OR: 1.64; 95% CI = 1.17–2.32	OR: 1.47; 95% CI = 1.03–2.09	OR: 1.40; 95% CI = 0.93–2.10	OR: 1.29; 95% CI = 0.85–1.95
Rockwood Frailty	-	OR: 1.04; 95% CI = 1.01–1.07*	-	OR: 1.04; 95% CI = 0.99–1.08*
Fried Frailty	-	OR: 1.78; 95% CI = 1.27–2.49*	-	OR: 1.49; 95% CI = 1.03–2.18*
Baseline cognitive score	-	-	OR: 0.72; 95% CI = 0.63–0.81	OR: 0.71; 95% CI = 0.62–0.81
Characteristic	Multivariable model 1	Multivariable model 2	Multivariable model 3	Multivariable model 4
Age	OR: 1.06; 95% CI = 1.03–1.09	OR: 1.05; 95% CI = 1.01–1.08	OR: 1.06; 95% CI = 1.02–1.09	OR: 1.04; 95% CI = 1.01–1.08
Sex	OR: 0.84; 95% CI = 0.46–1.53	OR: 0.79; 95% CI = 0.43–1.46	OR: 0.86; 95% CI = 0.43–1.72	OR: 0.83; 95% CI = 0.41–1.69
Stroke severity	OR: 1.04; 95% CI = 0.97–1.12	OR: 1.05; 95% CI = 0.98–1.13	OR: 0.98; 95% CI = 0.88–1.10	OR: 0.99; 95% CI = 0.88–1.12
Education (years)	OR: 0.92; 95% CI = 0.81–1.03	OR: 0.93; 95% CI = 0.83–1.04	OR: 0.97; 95% CI = 0.86–1.09	OR: 0.97; 95% CI = 0.87–1.09
Brain Frailty	-	OR: 1.47; 95% CI = 1.03–2.09	-	OR: 1.29; 95% CI = 0.85–1.95
Rockwood Frailty	OR: 1.05; 95% CI = 1.02–1.08	OR: 1.04; 95% CI = 1.01–1.07	OR: 1.04; 95% CI = 1.01–1.08	OR: 1.04; 95% CI = 0.99–1.08
Fried Frailty	-	-	-	-
Baseline cognitive score	-	-	OR: 0.70; 95% CI = 0.62–0.79	OR: 0.71; 95% CI = 0.62–0.81
Characteristic	Multivariable model 1	Multivariable model 2	Multivariable model 3	Multivariable model 4
Age	OR: 1.07; 95% CI = 1.04–1.10	OR: 1.05; 95% CI = 1.01–1.08	OR: 1.06; 95% CI = 1.03–1.10	OR: 1.04; 95% CI = 1.01–1.08
Sex	OR: 093; 95% CI = 0.50–1.71	OR: 0.86; 95% CI = 0.46–1.62	OR: 0.89; 95% CI = 0.44–1.80	OR: 0.86; 95% CI = 0.42–1.76
Stroke severity	OR: 1.04; 95% CI = 0.96–1.12	OR: 1.04; 95% CI = 0.96–1.13	OR: 0.99; 95% CI = 0.88–1.11	OR: 0.99; 95% CI = 0.89–1.12
Education (years)	OR: 0.90; 95% CI = 0.80–1.02	OR: 0.92; 95% CI = 0.82–1.04	OR: 0.96; 95% CI = 0.85–1.08	OR: 0.97; 95% CI = 0.86–1.09
Brain Frailty	-	OR: 1.48; 95% CI = 1.04–2.12	-	OR: 1.37; 95% CI = 0.91–2.07
Rockwood Frailty	-	-	-	-
Fried Frailty	OR: 1.93; 95% CI = 1.39–2.67	OR: 1.78; 95% CI = 1.27–2.49	OR: 1.57; 95% CI = 1.08–2.26	OR: 1.49; 95% CI = 1.03–2.18
Baseline cognitive score	-	-	OR: 0.72; 95% CI = 0.63–0.82	OR: 0.73; 95% CI = 0.64–0.82

Model 1 = Age, sex, stroke severity, education controlled for as covariates; Model 2 = model 1 + brain frailty or Rockwood frailty or Fried frailty (dependent on comparison) controlled for as covariates; Model 3 = model 1 + baseline cognitive score controlled for as covariates; Model 4 = model 2 plus baseline cognitive score controlled for as covariates.

* NB: Rockwood frailty and Fried frailty were entered into models 2 and 4 separately, they were not both included in the same regression model for either analysis. Output data are presented in one table for convenience.

Sensitivity analysis restricted to those with 18-month cognitive test data only was fully consistent with our primary results (Supplemental Materials S3).

### Subgroup analyses

Thirty-five participants had a pre-existing diagnosis of dementia. Subgroup analysis excluding participants with a formal diagnosis of dementia at baseline were fully consistent with our primary results (Supplementary Materials S4).

Forty-six participants had a TIA only. Subgroup analysis additionally excluding participants with a TIA were also consistent with our primary results.

## Discussion

Our findings suggest that brain frailty, Fried frailty, and Rockwood frailty are distinct, but frequently co-occurring constructs. Despite a weak overall correlation between measures, almost three-fourth of those who are frail according to the Rockwood frailty index, and >50% of those who were frail according to the Fried scale, had moderate to severe brain frailty scores and the prevalence of moderate to severe brain frailty increased as frailty status moved from robust to pre-frail to frail. Moreover, while prevalence of brain frailty in those who are frail appears substantial, it is also clear that each of our respective frailty measures retains an independent association with long-term cognitive impairment following stroke. Our findings are consistent with Wallace et al.^26,27^ suggesting frailty increases brain pathology but that it also plays a role in development of dementia above and beyond the brain pathogenesis.

Although brain frailty and Rockwood frailty were no longer significantly associated with long-term cognitive impairment after adjusting for baseline cognitive test performance, up to one-fourth of stroke survivors cannot fully complete traditional pen-and paper cognitive tests.^28^ Frailty measures may therefore be useful as alternate prognostic indicators of long-term cognitive impairment in these instances, although it should first be established if frailty is associated with long-term cognitive impairment independent of being untestable on baseline cognitive testing.

The Fried frailty phenotype was distinct from the two other frailty measures evaluated here in that it appears to have prognostic value independent of both imaging-based brain frailty and baseline cognitive test performance. Most stroke survivors show cognitive impairment in the immediate aftermath of stroke, but trajectories of recovery/further decline are variable. Measurement of Fried frailty may therefore be of value when evaluating cognitive prognosis. Fried frailty is also a potentially modifiable form of frailty^29^ and as such could be a target for interventions designed to minimise the long-term cognitive impact of a stroke. Physical and cognitive outcomes are often interrelated^30^; increased exercise has been associated with reduced white matter hyperintensities and cerebral atrophy in older adults,^31^ and physical activity plus risk factor reduction interventions have been shown to reduce rates of long-term cognitive decline.^32^ This suggests that long-term cognitive outcomes may be improved by addressing issues of physical function, such as frailty. However, studies aiming to treat symptoms of the Fried frailty phenotype have produced mixed results overall,^33^ and while symptoms of Fried frailty may be improved through exercise or nutritional therapies, this may do little to address the root cause of the syndrome. In addition, concerns have previously been raised regarding the feasibility of screening for Fried frailty in the acute stroke setting,^1^ thus establishing when and how to measure this condition in stroke also requires further exploration.

Rockwood frailty may be more measurable and there is some evidence^34^ that interventions emphasising the importance of nutrition, exercise and adherence to medication may be able to prevent further health decline. While Rockwood frailty likely cannot be reversed, prevention of further decline could be an important intervention for this population and may help to stop the risk of long-term cognitive issues from increasing further.

Whether brain frailty is treatable remains to be established. Previous studies^35^ have suggested that antihypertensive medications may help to slow the progression of white matter hyperintensities and that this may in part play a role in reducing risk of dementia. However, evidence is limited,^36^ hence this is an important avenue for further investigation.

### Strengths and limitations

Our study involved a highly inclusive stroke and TIA cohort, and we followed best practice guidance for conduct and reporting. We ensured blinding and double scoring to minimise risk of bias and conducted a series of subgroup analyses to ensure our results were translatable to clinical practice. Our population appears generalisable, and we used CT imaging data to generate brain frailty scores meaning our results will be applicable to most stroke services. Despite this, there are important limitations to note. Eighteen-month cognitive test data were unavailable for a proportion of our sample, meaning that outcome assessment was reliant upon clinical case notes and primary/secondary care medical records. Although reliable dementia diagnoses can still be achieved using this approach,^37^ some minor cases of cognitive impairment may have been missed; however, our sensitivity analysis suggests that this limitation did not significantly impact upon our results.

There were differences between risk of missing data and risk of in-study death based on pre-stroke frailty/brain frailty status. These differences may have impacted study significance values and effect sizes. However, this limitation is synonymous with longitudinal frailty and cognition research and may not be surmountable.

Our post hoc analyses were not powered to include the additional variables investigated; hence, the lack of significant predictive power of Rockwood frailty and brain frailty after including additional covariates could be related to lack of adequate statistical power.

Lastly, the challenges of operationalising frailty mean that the relative overlap between measures, along with the strength of relationships demonstrated in this study, may deviate if different frailty measures are employed – particularly if a larger cognitive component is adopted in respective physical frailty measures. However, the specific deficits included on the Rockwood and Mitnitski index are considered to be of less importance than the number of deficits measured,^38^ while Fried frailty also typically shows similar overall associations despite variations in methods of measurement.^39^ On this basis, we would generally expect the associations between frailty and long-term cognitive impairment observed in this study to hold.

## Conclusions

Evaluating the combination of pre-existing leukoaraiosis, atrophy and old infarcts via routinely collected CT scan images appears to be a valid method of establishing a person’s brain resilience or vulnerability following stroke. Many stroke survivors who show signs of frailty before their stroke also show signs of brain frailty. However, despite an apparent co-occurrence between brain frailty and traditional frailty, each respective measure retains independent value for predicting long-term adverse cognitive outcomes. Our findings emphasise the public health importance of life-course risk factors on the development of dementia,^40^ and suggest Fried frailty may be a viable target for interventions aiming to reduce post-stroke dementia risk.

## Supplemental Material

sj-docx-1-eso-10.1177_23969873231186480 – Supplemental material for Physical and brain frailty in ischaemic stroke or TIA: Shared occurrence and outcomes. A cohort study Click here for additional data file. Supplemental material, sj-docx-1-eso-10.1177_23969873231186480 for Physical and brain frailty in ischaemic stroke or TIA: Shared occurrence and outcomes. A cohort study by Martin Taylor-Rowan, Melanie Hafdi, Bogna Drozdowska, Emma Elliott, Joanna Wardlaw and Terence J Quinn in European Stroke Journal

## References

[1] Taylor-Rowan M Cuthbertson G Keir R , et al. The prevalence of frailty among acute stroke patients, and evaluation of method of assessment. Clin Rehabil 2019; 33: 1688–1696. 30971115 10.1177/0269215519841417

[2] Burton JK Stewart J Blair M , et al. Prevalence and implications of frailty in acute stroke: Systematic review & meta-analysis. Age Ageing 2022; 51: afac064. 10.1093/ageing/afac064 PMC9037368 35352795

[3] Joyce N Atkinson T Mc Guire K , et al. Frailty and stroke thrombectomy outcomes—an observational cohort study. Age Ageing 2022; 51: afab260. 10.1093/ageing/afab260 35150584

[4] Taylor-Rowan M Keir R Cuthbertson G , et al. Pre-stroke frailty is independently associated with post-stroke cognition: a cross-sectional study. J Int Neuropsychol Soc 2019; 25: 501–506. 30821222 10.1017/S1355617719000092

[5] Munthe-Kaas R Aam S Saltvedt I , et al. Is frailty index a better predictor than pre-stroke modified rankin scale for neurocognitive outcomes 3-months post-stroke? BMC Geriatr 2022; 22: 139. 35183106 10.1186/s12877-022-02840-y PMC8857811

[6] Clegg A Young J Iliffe S , et al. Frailty in elderly people. J Lancet 2013; 381: 752–762. 10.1016/S0140-6736(12)62167-9 PMC4098658 23395245

[7] Rockwood K Song X MacKnight C , et al. A global clinical measure of fitness and frailty in elderly people. CMAJ 2005; 173: 489–495. 16129869 10.1503/cmaj.050051 PMC1188185

[8] Fried LP Tangen CM Walston J , et al. Frailty in older adults: evidence for a phenotype. J Gerontol A Biol Sci Med Sci 2001; 56: M146–M156. 10.1093/gerona/56.3.m146 11253156

[9] Rockwood K Mitnitski AB MacKnight C . Some mathematical models of frailty and their clinical implications. Rev Clin Gerontol 2002; 12: 109–117.

[10] Schmidt R Petrovic K Ropele S , et al. Progression of leukoaraiosis and cognition. Stroke 2007; 38: 2619–2625. 17673724 10.1161/STROKEAHA.107.489112

[11] Appleton JP Woodhouse LJ Adami A , et al. Imaging markers of small vessel disease and brain frailty, and outcomes in acute stroke. Neurol 2020; 94: e439–e452. 10.1212/WNL.0000000000008881 PMC7080284 31882527

[12] KelaiditiE CesariM CanevelliM , et al. Cognitive frailty: rational and definition from an (i.A.N.A./i.A.G.G.) international consensus group. J Nutr Health Aging 2013; 17: 726–734.2415464210.1007/s12603-013-0367-2

[13] IST-3 Collaborative Group. Association between brain imaging signs, early and late outcomes, and response to intravenous alteplase after acute ischaemic stroke in the third international stroke trial (ist-3): Secondary analysis of a randomised controlled trial. Lancet Neurol 2015; 14: 485–496. 25819484 10.1016/S1474-4422(15)00012-5 PMC4513190

[14] Chen WT Chou KH Liu LK , et al. Reduced cerebellar gray matter is a neural signature of physical frailty. Hum Brain Mapp 2015; 36: 3666–3676. 26096356 10.1002/hbm.22870 PMC6869536

[15] Song X Mitnitski A Rockwood K . Age-related deficit accumulation and the risk of late-life dementia. Alzheimers Res Ther 2014; 6: 54. 25356088 10.1186/s13195-014-0054-5 PMC4212514

[16] von Elm E Altman DG Egger M , et al. Strengthening the reporting of observational studies in epidemiology (strobe) statement: Guidelines for reporting observational studies. BMJ 2007; 335: 806–808. 17947786 10.1136/bmj.39335.541782.AD PMC2034723

[17] Quinn TJ Taylor-Rowan M Elliott E , et al. Research protocol - assessing post-stroke psychology longitudinal evaluation (apple) study: a prospective cohort study in stroke. Cereb Circ Cogn Behav 2022; 3: 100042. 36324404 10.1016/j.cccb.2022.100042 PMC9616226

[18] Wardlaw JM Doubal F Brown R , et al. Rates, risks and routes to reduce vascular dementia (r4vad), a UK-wide multicentre prospective observational cohort study of cognition after stroke: protocol. Eur Stroke J 2021; 6: 89–101. 33817339 10.1177/2396987320953312 PMC7995325

[19] Searle SD Mitnitski A Gahbauer EA , et al. A standard procedure for creating a frailty index. BMC Geriatr 2008; 8: 24. 18826625 10.1186/1471-2318-8-24 PMC2573877

[20] Mahoney FI Barthel DW . Functional evaluation: the barthel index. Md State Med J 1965; 14: 61–65. 14258950

[21] Lawton MP Brody EM . Assessment of older people: self-maintaining and instrumental activities of daily living. Gerontol 1969; 9: 179–186. 5349366

[22] Cesari M Demougeot L Boccalon H , et al. A self-reported screening tool for detecting community-dwelling older persons with frailty syndrome in the absence of mobility disability: the find questionnaire. PLoS One 2014; 9: e101745. 24999805 10.1371/journal.pone.0101745 PMC4084999

[23] American Psychiatric Association. Diagnostic and statistical manual of mental disorders: DSM-5. 5th ed. Washington, D.C: American Psychiatric Publishing, 2013.

[24] Swinscow TDV . Correlation and regression. In: MJ Campbell (ed.) Statistics at square one. London: BMJ Publishing Group, 1997; 75–85.

[25] Kohn MJS . Sample size calculators [website]. 2021.

[26] Wallace LMK Theou O Godin J , et al. Investigation of frailty as a moderator of the relationship between neuropathology and dementia in Alzheimer’s disease: a cross-sectional analysis of data from the rush memory and aging project. Lancet Neurol 2019; 18: 177–184. 30663607 10.1016/S1474-4422(18)30371-5 PMC11062500

[27] Wallace L Hunter S Theou O , et al. Frailty and neuropathology in relation to dementia status: the cambridge city over-75s cohort study. Int Psychogeriatr 2021; 33: 1035–1043. 33586645 10.1017/S1041610220003932

[28] Elliott E Drozdowska BA Taylor-Rowan M , et al. Who is classified as untestable on brief cognitive screens in an acute stroke setting? Diagnostics 2019; 9: 95. 31416176 10.3390/diagnostics9030095 PMC6787589

[29] Walston J Buta B Xue QL . Frailty screening and interventions: considerations for clinical practice. Clin Geriatr Med 2018; 34: 25–38. 29129215 10.1016/j.cger.2017.09.004 PMC5726589

[30] McHutchison CA Cvoro V Makin S , et al. Functional, cognitive and physical outcomes 3 years after minor lacunar or cortical ischaemic stroke. J Neurol Neurosurg Psychiatry 2019; 90: 436–443. 30554134 10.1136/jnnp-2018-319134 PMC6581154

[31] Gow AJ Bastin ME Muñoz Maniega S , et al. Neuroprotective lifestyles and the aging brain: activity, atrophy, and white matter integrity. Neurology 2012; 79: 1802–1808. 23091073 10.1212/WNL.0b013e3182703fd2

[32] Ngandu T Lehtisalo J Solomon A , et al. A 2 year multidomain intervention of diet, exercise, cognitive training, and vascular risk monitoring versus control to prevent cognitive decline in at-risk elderly people (finger): a randomised controlled trial. J Lancet 2015; 385: 2255–2263. 10.1016/S0140-6736(15)60461-5 25771249

[33] Travers J Romero-Ortuno R Bailey J , et al. Delaying and reversing frailty: a systematic review of primary care interventions. Br J Gen Pract 2019; 69: e61–e69. 10.3399/bjgp18X700241 PMC6301364 30510094

[34] Ekdahl AW Alwin J Eckerblad J , et al. Long-term evaluation of the ambulatory geriatric assessment: a frailty intervention trial (age-fit): clinical outcomes and total costs after 36 months. J Am Med Dir Assoc 2016; 17: 263–268. 26805750 10.1016/j.jamda.2015.12.008

[35] Lai Y Jiang C Du X , et al. Effect of intensive blood pressure control on the prevention of white matter hyperintensity: systematic review and meta-analysis of randomized trials. J Clin Hypertens 2020; 22: 1968–1973. 10.1111/jch.14030 PMC8029786 33459521

[36] Wardlaw JM Debette S Jokinen H , et al. Eso guideline on covert cerebral small vessel disease. Eur Stroke J 2021; 6: IV. 34414305 10.1177/23969873211027002 PMC8370062

[37] Wilkinson T Ly A Schnier C , et al. Identifying dementia cases with routinely collected health data: a systematic review. Alzheimer Dement 2018; 14: 1038–1051. 10.1016/j.jalz.2018.02.016 PMC6105076 29621480

[38] Rockwood K Mitnitski A . Frailty defined by deficit accumulation and geriatric medicine defined by frailty. Clin Geriatr Med 2011; 27: 17–26. 21093719 10.1016/j.cger.2010.08.008

[39] Papachristou E Wannamethee SG Lennon LT , et al. Ability of self-reported frailty components to predict incident disability, falls, and all-cause mortality: results from a population-based study of older British men. J Am Med Dir Assoc 2017; 18: 152–157. 27742583 10.1016/j.jamda.2016.08.020 PMC5270459

[40] Livingston G Huntley J Sommerlad A , et al. Dementia prevention, intervention, and care: 2020 report of the lancet commission. J Lancet 2020; 396: 413–446. 10.1016/S0140-6736(20)30367-6 PMC7392084 32738937

