# Supplementary material for: Physical and brain frailty in ischaemic stroke or TIA: Shared occurrence and outcomes. A cohort study
Source: Eur Stroke J. 2023 Jul 7;8(4):1011–20. doi: 10.1177/23969873231186480 (PMC10683729; doi:10.1177/23969873231186480)
Supplement: sj-docx-1-eso-10.1177_23969873231186480 – Supplemental material for Physical and brain frailty in ischaemic stroke or TIA: Shared occurrence and outcomes. A cohort study [file sj-docx-1-eso-10.1177_23969873231186480.docx]

**Supplementary materials**

**Contents:**

**Supplementary materials S1**. Template used for brain frailty scoring

**Supplementary materials S2:** Rockwood accumulated deficits measurement scale

**Supplementary materials S3:** Sensitivity analysis restricted to participants with 18 month cognitive data available

**Supplementary materials S4:** Regression output of subgroup analyses

**Supplmentary materils Figure S1**. Template used for guiding brain frailty scoring

**
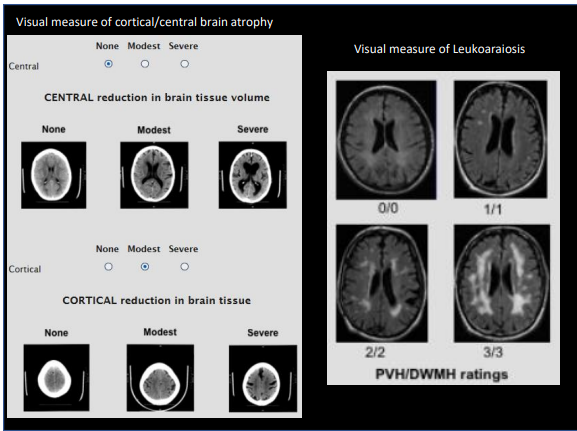
**

**Supp materials Figure S2:** Rockwood accumulated deficits measurement scale

| **Frailty Index** | | |
| --- | --- | --- |
| Mood disorder  Hypertension  Atrial Fibrilation  Previous cerebrovascular disease  Diabetes  Vascular disease  Hyperlipidaemia  Heart failure  Liver disease  Peptic ulcer  Arthritis | Care-home resident  Carers  Hearing impairment  Visual impairment  Falls (>2 in last year)  COPD  Cancer  Renal failure  Migraine  Seizure disorder  Hx of delirium | Mobility aid  Assistance walking  Problems getting dressed  Problems bathing  Problems carrying out personal grooming  Family Hx of dementia  Problems cooking  Problems going out alone  Continence bladder  Continence bowel |

**Supplementary materials S3:** Sensitivity analysis restricted to participants with 18 month cognitive data available

| **Characteristic** | **Odds ratios** |
| --- | --- |
| Age | 1.042 (95%CI=0.99-1.10) |
| Sex | 0.55 (95%CI=0.19-1.53) |
| Stroke severity | 1.04 (95%CI=0.91-1.19) |
| Education (years) | 1.02 (95%CI=0.85-1.24) |
| Brain Frailty | 1.78 (95%CI=1.02-3.09) |
| **Characteristic** | **Odds ratios** |
| Age | 1.07 (95%CI=1.02-1.13) |
| Sex | 0.71 (95%CI=0.27-1.90) |
| Stroke severity | 0.98 (95%CI=0.84-1.14) |
| Education (years) | 0.97 (95%CI=0.79-1.18) |
| Rockwood Frailty | 1.07 (95%CI=1.02-1.13) |
| **Characteristic** | **Odds ratios** |
| Age | 1.08 (95%CI=1.02-1.14) |
| Sex | 0.81 (95%CI=0.30-2.19) |
| Stroke severity | 1.01 (95%CI=0.88-1.16) |
| Education (years) | 0.98 (95%CI=0.81-1.19) |
| Fried Frailty | 1.82 (95%CI=1.12-2.95) |

**Supplementary materials S4:** Subgroup analyses

**Baseline Dementia removed:**

***Brain frailty regression output for association with 18-month cognition***

| **Characteristic** | **Multivariable model 1** | **Multivariable model 2** | **Multivariable model 3** | **Multivariable model 4** |
| --- | --- | --- | --- | --- |
| Age | OR:1.04 95%CI=1.00-1.07 | OR:1.04 95%CI=1.00-1.07 | OR:1.04 95%CI=1.00-1.08 | OR:1.04 95%CI=1.00-1.08 |
| Sex | OR:0.71 95%CI=0.38-1.33 | OR:0.74 95%CI=0.40-1.40 | OR:0.76 95%CI=0.37-1.56 | OR:0.80 95%CI=0.39-1.64 |
| Stroke severity | OR:1.07 95%CI=0.99-1.15 | OR:1.05 95%CI=0.98-1.13 | OR:1.02 95%CI=0.91-1.15 | OR:1.00 95%CI=0.89-1.13 |
| Education (years) | OR:0.92 95%CI=0.82-1.03 | OR:0.93 95%CI=0.82-1.05 | OR:0.96 95%CI=0.85-1.08 | OR:0.97 95%CI=0.86-1.09 |
| Brain Frailty | OR:1.71 95%CI=1.19-2.44 | OR:1.53 95%CI=1.06-2.22 | OR:1.44 95%CI=0.95-2.17 | OR:1.34 95%CI=0.88-2.04 |
| Rockwood Frailty | - | OR:1.04 95%CI=1.003-1.07 | - | OR:1.03 95%CI=0.99-1.07 |
| Fried Frailty | - | OR:1.85 95%CI=1.31-2.61 | - | OR:1.56 95%CI=1.07-2.29 |
| Baseline cognitive score | - | - | OR:0.74 95%CI=0.65-0.83 | OR:0.73 95%CI=0.64-0.83 |

***NB: Rockwood frailty and Fried frailty were entered into models 2 and 4 separately, they were not both included in the same regression model for either analysis. Output data are presented in one table for convenience.**

***Rockwood frailty regression output for association with 18-month cognition***

| **Characteristic** | **Multivariable model 1** | **Multivariable model 2** | **Multivariable model 3** | **Multivariable model 4** |
| --- | --- | --- | --- | --- |
| Age | OR:1.06 95%CI=1.03-1.09 | OR:1.04 95%CI=1.00-1.07 | OR:1.06 95%CI=1.02-1.09 | OR:1.04 95%CI=1.00-1.08 |
| Sex | OR:0.80 95%CI=0.44-1.49 | OR:0.74 95%CI=0.40-1.40 | OR:0.83 95%CI=0.41-1.69 | OR:0.80 95%CI=0.39-1.64 |
| Stroke severity | OR:1.04 95%CI=0.97-1.12 | OR:1.05 95%CI=0.98-1.13 | OR:0.99 95%CI=0.88-1.11 | OR:1.00 95%CI=0.89-1.13 |
| Education (years) | OR:0.92 95%CI=0.81-1.03 | OR:0.93 95%CI=0.82-1.05 | OR:0.96 95%CI=0.85-1.09 | OR:0.97 95%CI=0.86-1.09 |
| Brain Frailty | - | OR:1.53 95%CI=1.06-2.22 | - | OR:1.34 95%CI=0.88-2.04 |
| Rockwood Frailty | OR:1.05 95%CI=1.02-1.08 | OR:1.04 95%CI=1.003-1.07 | OR:1.04 95%CI=1.00-1.08 | OR:1.03 95%CI=0.99-1.07 |
| Fried Frailty | - | - | - | - |
| Baseline cognitive score | - | - | OR:0.72 95%CI=0.63-0.81 | OR:0.73 95%CI=0.64-0.83 |

***Fried frailty regression output for association with 18-month cognition***

| **Characteristic** | **Multivariable model 1** | **Multivariable model 2** | **Multivariable model 3** | **Multivariable model 4** |
| --- | --- | --- | --- | --- |
| Age | OR:1.06 95%CI=1.03-1.09 | OR:1.04 95%CI=1.00-1.07 | OR:1.06 95%CI=1.02-1.09 | OR:1.04 95%CI=0.99-1.08 |
| Sex | OR:0.88 95%CI=0.47-1.67 | OR:0.81 95%CI=0.42-1.56 | OR:0.86 95%CI=0.42-1.77 | OR:0.82 95%CI=0.40-1.71 |
| Stroke severity | OR:1.03 95%CI=0.95-1.12 | OR:1.04 95%CI=0.96-1.13 | OR:0.99 95%CI=0.89-1.12 | OR:1.00 95%CI=0.89-1.13 |
| Education (years) | OR:0.90 95%CI=0.80-1.03 | OR:0.92 95%CI=0.82-1.04 | OR:0.95 95%CI=0.83-1.08 | OR:0.96 95%CI=0.85-1.09 |
| Brain Frailty | - | OR:1.56 95%CI=1.07-2.26 | - | OR:1.42 95%CI=0.93-2.17 |
| Rockwood Frailty | - | - | - | - |
| Fried Frailty | OR:1.99 95%CI=1.43-2.79 | OR:1.85 95%CI=1.31-2.61 | OR:1.63 95%CI=1.13-2.37 | OR:1.56 95%CI=1.07-2.29 |
| Baseline cognitive score | - | - | OR:0.74 95%CI=0.65-0.84 | OR:0.75 95%CI=0.66-0.85 |

**Model1=Age, sex, stroke severity, education controlled for as covariates; Model 2= model 1+brain frailty or Rockwood frailty or Fried frailty (dependent on comparison) controlled for as covariates; Model 3=model 1+ baseline cognitive score controlled for as covariates; Model 4=model2 plus baseline cognitive score controlled for as covariates.**

**Baseline Dementia and TIA removed:**

***Brain frailty regression output for association with 18-month***

| **Characteristic** | **Multivariable model 1** | **Multivariable model 2** | **Multivariable model 3** | **Multivariable model 4** |
| --- | --- | --- | --- | --- |
| Age | OR:1.03 95%CI=0.99-1.07 | OR:1.03 95%CI=0.99-1.07 | OR:1.04 95%CI=0.99-1.09 | OR:1.04 95%CI=0.99-1.09 |
| Sex | OR:0.81 95%CI=0.39-1.69 | OR:0.83 95%CI=0.40-1.75 | OR:0.80 95%CI=0.34-1.88 | OR:0.83 95%CI=0.35-1.96 |
| Stroke severity | OR:1.09 95%CI=1.01-1.18 | OR:1.07 95%CI=0.99-1.17 | OR:1.07 95%CI=0.94-1.22 | OR:1.05 95%CI=0.92-1.19 |
| Education (years) | OR:0.89 95%CI=0.77-1.03 | OR:0.90 95%CI=0.77-1.04 | OR:0.92 95%CI=0.78-1.09 | OR:0.92 95%CI=0.78-1.10 |
| Brain Frailty | OR:1.99 95%CI=1.30-3.08 | OR:1.79 95%CI=1.15-2.79 | OR:1.59 95%CI=0.96-2.63 | OR:1.46 95%CI=0.88-2.43 |
| Rockwood Frailty | - | OR:1.04 95%CI=0.99-1.08 | - | OR:1.04 95%CI=0.99-1.09 |
| Fried Frailty | - | OR:1.82 95%CI=1.22-2.71 | - | OR:1.82 95%CI=1.16-2.89 |
| Baseline cognitive score | - | - | OR:0.73 95%CI=0.63-0.84 | OR:0.71 95%CI=0.61-0.83 |

***NB: Rockwood frailty and Fried frailty were entered into models 2 and 4 separately, they were not both included in the same regression model for either analysis. Output data are presented in one table for convenience.**

***Rockwood frailty regression output for association with 18-month***

| **Characteristic** | **Multivariable model 1** | **Multivariable model 2** | **Multivariable model 3** | **Multivariable model 4** |
| --- | --- | --- | --- | --- |
| Age | OR:1.06 95%CI=1.02-1.10 | OR:1.03 95%CI=0.99-1.07 | OR:1.06 95%CI=1.02-1.10 | OR:1.04 95%CI=0.99-1.09 |
| Sex | OR:0.86 95%CI=0.42-1.77 | OR:0.83 95%CI=0.40-1.75 | OR:0.83 95%CI=0.35-1.93 | OR:0.83 95%CI=0.35-1.96 |
| Stroke severity | OR:1.06 95%CI=0.98-1.14 | OR:1.07 95%CI=0.99-1.17 | OR:1.03 95%CI=0.91-1.17 | OR:1.05 95%CI=0.92-1.19 |
| Education (years) | OR:0.88 95%CI=0.75-1.02 | OR:0.90 95%CI=0.77-1.04 | OR:0.92 95%CI=0.77-1.09 | OR:0.92 95%CI=0.78-1.10 |
| Brain Frailty | - | OR:1.79 95%CI=1.15-2.79 | - | OR:1.46 95%CI=0.88-2.43 |
| Rockwood Frailty | OR:1.05 95%CI=1.01-1.09 | OR:1.04 95%CI=0.99-1.08 | OR:1.05 95%CI=1.00-1.09 | OR:1.04 95%CI=0.99-1.09 |
| Fried Frailty | - | - | - | - |
| Baseline cognitive score | - | - | OR:0.70 95%CI=0.60-0.81 | OR:0.71 95%CI=0.61-0.83 |

***Fried frailty regression output for association with 18-month***

| **Characteristic** | **Multivariable model 1** | **Multivariable model 2** | **Multivariable model 3** | **Multivariable model 4** |
| --- | --- | --- | --- | --- |
| Age | OR:1.06 95%CI=1.03-1.10 | OR:1.03 95%CI=0.99-1.07 | OR:1.06 95%CI=1.02-1.10 | OR:1.04 95%CI=0.99-1.09 |
| Sex | OR:0.93 95%CI=0.44-1.97 | OR:0.90 95%CI=0.42-1.94 | OR:0.85 95%CI=0.36-2.00 | OR:0.86 95%CI=0.36-2.06 |
| Stroke severity | OR:1.05 95%CI=0.97-1.15 | OR:1.07 95%CI=0.98-1.17 | OR:1.04 95%CI=0.91-1.18 | OR:1.06 95%CI=0.93-1.20 |
| Education (years) | OR:0.86 95%CI=0.73-1.01 | OR:0.89 95%CI=0.76-1.04 | OR:0.89 95%CI=0.74-1.07 | OR:0.91 95%CI=0.76-1.09 |
| Brain Frailty | - | OR:1.84 95%CI=1.16-2.89 | - | OR:1.60 95%CI=0.95-2.69 |
| Rockwood Frailty | - | - | - | - |
| Fried Frailty | OR:2.00 95%CI=1.37-2.93 | OR:1.82 95%CI=1.22-2.71 | OR:1.67 95%CI=1.08-2.58 | OR:1.58 95%CI=1.01-2.48 |
| Baseline cognitive score | - | - | OR:0.72 95%CI=0.62-0.83 | OR:0.73 95%CI=0.63-0.84 |

**Model1=Age, sex, stroke severity, education controlled for as covariates; Model 2= model 1+brain frailty or Rockwood frailty or Fried frailty (dependent on comparison) controlled for as covariates; Model 3=model 1+ baseline cognitive score controlled for as covariates; Model 4=model2 plus baseline cognitive score controlled for as covariates.**
